# Genetic Risk Prediction of COVID-19 Susceptibility and Severity in the Indian Population

**DOI:** 10.3389/fgene.2021.714185

**Published:** 2021-10-11

**Authors:** P. Prakrithi, Priya Lakra, Durai Sundar, Manav Kapoor, Mitali Mukerji, Ishaan Gupta, The Indian Genome Variation Consortium

**Affiliations:** ^1^ Genomics and Molecular Medicine, CSIR Institute of Genomics and Integrative Biology, New Delhi, India; ^2^ Department of Biochemical Engineering and Biotechnology, Indian Institute of Technology Delhi, New Delhi, India; ^3^ Department of Neuroscience, Icahn School of Medicine at Mt. Sinai, New York, NY, United States

**Keywords:** COVID-19, Indian population, polygenic risk score, genetics, suscepibility, genetic risk prediction

## Abstract

Host genetic variants can determine their susceptibility to COVID-19 infection and severity as noted in a recent Genome-wide Association Study (GWAS). Given the prominent genetic differences in Indian sub-populations as well as differential prevalence of COVID-19, here, we compute genetic risk scores in diverse Indian sub-populations that may predict differences in the severity of COVID-19 outcomes. We utilized the top 100 most significantly associated single-nucleotide polymorphisms (SNPs) from a GWAS by Pairo-Castineira et al*.* determining the genetic susceptibility to severe COVID-19 infection, to compute population-wise polygenic risk scores (PRS) for populations represented in the Indian Genome Variation Consortium (IGVC) database. Using a generalized linear model accounting for confounding variables, we found that median PRS was significantly associated (*p* < 2 x 10^−16^) with COVID-19 mortality in each district corresponding to the population studied and had the largest effect on mortality (regression coefficient = 10.25). As a control we repeated our analysis on randomly selected 100 non-associated SNPs several times and did not find significant association. Therefore, we conclude that genetic susceptibility may play a major role in determining the differences in COVID-19 outcomes and mortality across the Indian sub-continent. We suggest that combining PRS with other observed risk-factors in a Bayesian framework may provide a better prediction model for ascertaining high COVID-19 risk groups and to design more effective public health resource allocation and vaccine distribution schemes.

## Introduction

Susceptibility to immune reaction-mediated diseases and viral infections are both observed to be heritable traits, and are associated with specific genetic variants (Kenney et al., 2017; Ellinghaus et al., 2020; Shelton et al., 2020; Kwok et al., 2021; Pairo-Castineira et al., 2021). The GWAS by Pairo-Castineira et al. in critically ill COVID-19 patients from a UK cohort identified strong genetic signals, related to antiviral defence mechanisms and inflammatory organ damage, that are potentially associated with COVID-19 severity. Among the top eight robust associations identified in the GWAS (Pairo-Castineira et al., 2021), two SNPs, namely, rs10735079 and rs2109069 are also present in the Indian Genome Variation Consortium (IGVC). The IGVC was a large-scale comprehensive study of the Indian sub-populations that was conducted to shed light on the genetic diversity among geographically and ethnically diverse Indian sub-populations. This study had identified a high degree of genetic distinctness, with respect to SNPs, in different Indian sub-populations (Indian Genome Variation Consortium, 2005; Indian Genome Variation Consortium, 2008). With the increasing number of COVID-19 cases and the evolving variants of SARS-CoV2 in India, a populous and a genetically diverse country, prioritizing vulnerable populations for COVID-19 vaccination is critical, given the limited production of vaccines and identification of genetic risk estimates associated with COVID-19 susceptibility can be beneficial in identifying susceptible population(s).

Genome-wide association studies have identified the genetic underpinnings of several diseases, and these variants together weighted by their effect sizes yield estimates for polygenic risk score (PRS). PRS provides an estimate of the genetic propensity of an individual to develop a disease and/or a trait (Chatterjee et al., 2016; Lewis and Vassos, 2020). Transethnic replication of GWAS effect sizes has been employed previously, however, it is challenging and might not lead to accurate predictions when applied to non-discovery GWAS populations, owing to biological differences, such as different patterns of linkage disequilibrium (LD), allele frequencies, and gene-environment interactions, in different populations (Novembre and Barton, 2018) and/or technical differences. For example, there will be no transethnic replication if there is significant difference in the LD structure across different ethnic populations (Martin et al., 2017). However, it has been shown that using training data sets that include samples from the discovery population in which the GWAS was conducted (confers the advantage of large sample size in the GWAS) as well as samples from the target population in which the PRS is aimed to be calculated (advantage of being the same ancestry), improves the prediction accuracy of the PRS (Li and Keating, 2014; Márquez-Luna et al., 2017). Hence using the causal variants identified in a discovery GWAS that overlap with the target population and not the SNPs in LD, and those with a conserved LD pattern across the discovery and target populations (Piffer, 2021), would improve the accuracy of PRS calculated in the target population using the effect sizes of corresponding SNPs from the discovery GWAS (Wang et al., 2020). Earlier studies also report association of observable traits with polygenic scores (Piffer, 2013; Piffer, 2015; Piffer, 2019).

Here, with prior information from the data of stratified Indian sub-populations, we calculated the PRSs with an aim to explore and identify Indian sub-populations that could be at a higher risk for COVID-19-mediated mortality. Considering the challenges associated with the transferability of the effect sizes, we also analyzed the differences in the patterns of LD, and used the SNPs with similar LD patterns in the discovery population and Indian population to ensure good prediction accuracy of the PRS. Based on these PRSs, we evaluated the population-wise susceptibility that can be of potential utility in more effective vaccine distribution schemes among Indian sub-populations.

## Materials and Methods

### Study Populations and Datasets

Summary statistics for genetic variants was obtained from a GWAS in 2,244 critically ill patients from 208 intensive care units, a majority of them of European Ancestry (∼75%), ∼11% of South Asian, 8% African and 7% of East Asian ancestries (Pairo-Castineira et al., 2021). The study had identified genetic signals related to host antiviral defense pathways and those mediating inflammatory organ damage in critical COVID-19 patients using Mendelian randomization, GWAS and transcriptome-wide association studies. DNA samples were genotyped using Illumina Global Screening Array v.3.0 + multi-disease bead chips (GSAMD-24v3-0-EA) and Infinium chemistry (Pairo-Castineira et al., 2021). Further, genotype data of 390 samples across 25 populations from the IGVC (Phase 3) were used for analyzing the Indian sub-populations. Briefly, they represent diverse ethno-linguistic and geographical regions of India, and house information about genome-wide SNPs across Indian populations. The data span four major linguistic lineages - Indo-European (IE), Dravidian (DR), Austro-Asiatic (AA), and Tibeto Burman (TB) from different geographic locations (north, south, east, west, and central) from contrasting ethnic backgrounds and ethnicity sub-categorized as caste groups (LP), religious groups (SP), and tribal isolated populations (IP) (Supplementary Table S1; Indian Genome Variation Consortium, 2005; Indian Genome Variation Consortium, 2008). Clusters of representative populations were identified through extensive analysis of a larger sample set of more than 2000 samples from 55 populations. Samples collected from these groups were genotyped on an OMNI array, Illumina Inc. (unpublished data) as a representation of Indian genomic diversity. The 25 populations of the IGVC used for this study map to 22 districts in India, for which COVID-19 mortality data was collected from official sites and publicly available repositories including https://www.covid19india.org/, https://covid19.Assam.gov.in/district/,https://api.covid19india.org/ (Supplementary Table S1).

### Polygenic Risk Score Calculation and Population Susceptibility

**FIGURE 1:**
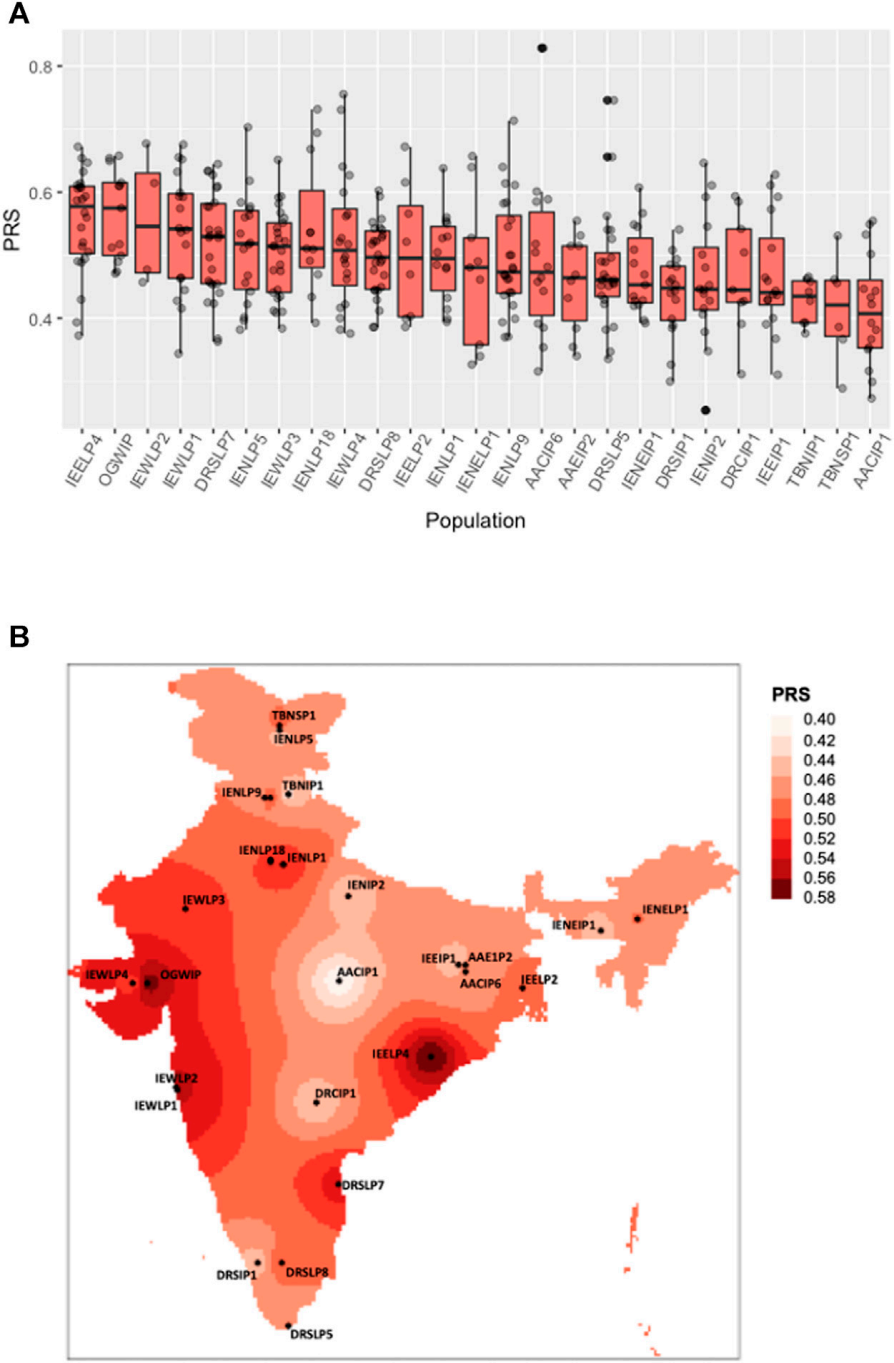
Distribution of polygenic risk scores across Indian sub-populations. **(A)** The boxplot shows a polygenic risk score distribution across 25 Indian sub-populations from the IGVC divided on the basis of linguistic and geographical regions (IP, tribal populations; LP, caste; and SP, religious groups). **(B)** Spatial distribution of PRSs calculated for IGVC populations spanning different districts of India.

The GWAS we use for our analysis identified numerous independent genome-wide significant SNPs for different ancestral populations, 75% of which were of European ancestry. These SNPs were overlapped with the IGVC data to identify common variants which were then sorted, filtered and selected on the basis of GWAS p*-*values (below the genome-wide threshold *p* < 10^−06^). The top 100 such SNPs from the study represented in the IGVC data were analysed for and ascertained to have similar LD patterns (for applicability of effect sizes from non-SAS ancestry) across the Indian sub-populations and the GWAS discovery population and were used for polygenic score analysis. The effect sizes of 30 SNPs were derived from Europeans, 40 from South Asian, 21 from East-Asian and seven from African ancestries. PRS of each individual was calculated using PLINKv1.9 (Purcell et al., 2007), and PRS for a population was calculated by taking the median PRS of all the individuals in that population. Population wise statistical significance was calculated using one-way ANOVA. The distribution of the PRS in the individuals across different IGVC populations was plotted using an R script as follows: For the spatial map showing PRS distribution (Figure 1B) we used an IDW algorithm and a tutorial from https://mgimond.github.io/Spatial/interpolation-in-r.html, licensed under a Creative Commons Attribution-NonCommercial 4.0 International License was referred to. The PRS for each district calculated, was multiplied with the corresponding population to calculate the potentially susceptible population. The districts/regions at a higher risk for the trait studied were also identified.

### Addressing Linkage Disequilibrium

While applying effect sizes derived from one ancestral population to another to calculate PRS, the accuracy is better when SNPs with similar LD patterns or allele frequencies between the discovery (majority of Europeans) and target (Indian) populations are used. Directly comparing LD patterns between Europeans (CEU) and IGVC populations was not possible because of the smaller number of SNPs represented in the OMNI array of the IGVC. So we first overlapped the GWAS results with the IGVC SNP data, from which the top 100 significant SNPs (sorted by p-value) were selected and verified for LD conservation. The genetic closeness of individuals of the IGVC to the SAS populations of the 1,000 Genomes has been shown in a recent study (Narang et al., 2020). Another study (Sengupta et al., 2016) has also displayed similar inferences with four distinct Indian ancestral categories - Ancestral North Indian, Ancestral South Indian, Austro-Asiatic and Tibeto Burman as represented by IE, DR, AA, and TB in the IGVC. Hence, one of the South Asian populations from the 1,000 Genomes was used to represent the Indian population (IGVC) for LD analysis, since the array data does not cover enough SNPs to compare with other global populations. The CEU (Utah residents (CEPH) with Northern and Western European ancestry), CHB (Han Chinese in Beijing - representative of East Asian ancestry), YRI (Yoruba in Ibadan, Nigeria—representative of African ancestry) and ITU (Indian Telugu from the UK - representative of the Indian ancestry) populations from the 1,000 Genomes project (Phase 3 release) were utilized to compare the patterns of LD (ftp://ftp-trace.ncbi.nih.gov/1000genomes/ftp/release/20130502/).

The LD pattern 5 MB around the top 100 most significantly associated SNPs were compared between each of these non-Indian ancestral populations with ITU using the varLD (v1.0) tool–a tool to compare the extent of LD differentiation at loci between pairwise populations, to assess LD structure across the discovery GWAS population and the target Indian population. (Ong and Teo, 2010). The minor allele frequencies for some of these SNPs were checked for ITU versus the other representative populations (CEU, YRI and CHB) from the Ensembl Genome browser (Howe et al., 2021) and with wANNOVAR (Chang and Wang, 2012). Many of them had similar frequencies across the populations. However, we did not necessarily apply the similar allele frequency criterion for the SNP selection, since LD conservation was seen for all the 100 SNPs. (Results section and Supplementary Figure S1).

### Modelling COVID-19 Mortality

The district level COVID-19 information till 2nd September, 2021 for those mapping to the 25 populations studied were collected from the publicly available sources as specified in the ‘Study populations and datasets’ section. A generalized linear model (GLM) was fit for deaths per million population of each district due to COVID-19 and PRS of the corresponding district. To account for potential confounders, we added percentage of population above 45 years of age, and sex ratio (number of females for every 1,000 males) to the GLM. Poisson distribution was used with the respective population of each district as an offset to control for overdispersion. The data were collected from the census records of India (https://censusindia.gov.in). A similar model was constructed for the data with the IENLP1 population removed as this was seen as an outlier due to a very high value for deaths/million. The pseudo R^2^ values were calculated as (model$null.deviance-model$deviance)/model$null.deviance (Windmeijer and Cameron, 1996). The results were then compared with the model fit earlier.

We further investigated whether the PRSs calculated from effect sizes of non-risk SNPs could have any effect on COVID-19 mediated mortality. For this, we selected 1,000 sets of 100 random SNPs that were not significantly associated with the trait from the same GWAS, and the GLM analyses were performed on each of the 1,000 datasets.

## Results

The polygenic predictors used in the present study were derived from Pairo-Castineira et al. (Pairo-Castineira et al., 2021), and applied on 25 geographically and ethnically diverse sub-populations of the Indian sub-continent (Indian Genome Variation Consortium, 2005; Indian Genome Variation Consortium, 2008). VarLD analysis indicated that almost all the 100 SNPs lie below the threshold (Supplementary Figure S1), suggesting that the LD structure was maintained between the discovery GWAS and target Indian populations. As shown in Figures 1A,B; Supplementary Table S1, we found a varying distribution of PRS in different sub-populations of India (one-way ANOVA, F (24, 365) = 3.072, *p* = 2.95 ✕ 10^−06^). Based on the PRS for each district, the susceptible population, i.e., the number of individuals in a population at risk for developing severe illness when infected with SARS-CoV-2 was also evaluated (Supplementary Table S1).

**FIGURE 2:**
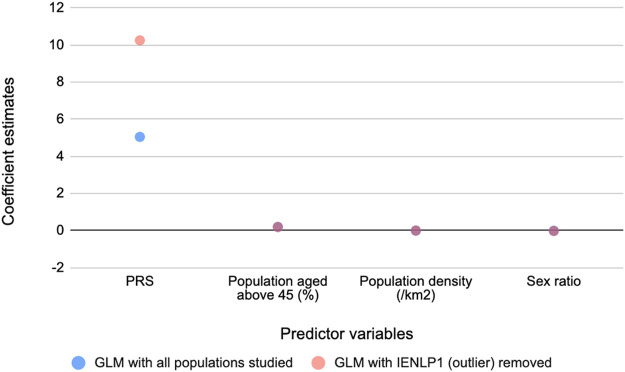
Coefficient estimates of the predictor variables for COVID-19 mediated mortality from the GLM models. PRS has the strongest effect on mortality.

To determine the relationship between these PRSs and COVID-19 mortality, generalized linear regression analysis was performed. The GLMs suggested that PRS has the strongest significant effect (*p* < 2 x 10^−16^) on mortality while the potential confounders considered had significant (*p* < 2 x 10^−16^) but negligible effects. The same trend was observed on removing an outlier population, IENLP1 and the coefficient estimate for PRS had improved two-fold and the fit improved (pseudo R^2^ = 0.46) (Table 1). Based on the coefficient estimates (regression coefficient = 10.25), the average deaths per million population of the district would significantly increase by 10.25 units with increase in PRS by 1 unit. The coefficients of age and population density are negligible (effect of population density is not significant for the GLM that included all data points). Sex ratio (number of females for every 1,000 males) has a significant but negligible negative effect. (Figure 2; Table 1).

**TABLE 1:** Results of the GLMs constructed.

Predictor variables	All data (deaths/million ∼ predictor variables) Pseudo R^2^ = 0.07				1 outlier IENLP1 (deaths/million = 4200) removed (deaths/million ∼ predictor variables) Pseudo R^2^ = 0.46			
	Estimate	Std.Error	Z value	Pr (>|z|)	Estimate	Std.Error	Z value	Pr (>|z|)
(Intercept)	−7.130	0.195	−36.512	<2 x 10^−16^	−3.340	0.196	−17.020	<2 x 10^−16^
PRS	5.050	0.290	17.410	<2 x 10^−16^	10.250	0.403	25.480	<2 x 10^−16^
Population above 45 years of age (%)	0.200	0.004	53.761	<2 x 10^−16^	0.190	0.004	48.540	<2 x 10^−16^
Population density (/km^2^)	0.000	0.000	−1.366	0.172	0.000	0.000	36.310	<2 x 10^−16^
Sex ratio	−0.010	0.000	−34.941	<2 x 10^−16^	−0.020	0.000	−100.470	<2 x 10^−16^

No significant association of PRS derived from non-risk SNPs with the COVID-19 mortality was observed in the GLMs as shown in Supplementary Figure S2. Our results indicate a significant association of PRS with the number of COVID-19-related deaths, and thereby can provide support for population specific prioritization in COVID-19 vaccination program. For example, the IEWLP1 population, displays higher PRS as well as high covid-19 mediated mortality. This population maps to Raigad district in Maharashtra which has a high population density (369.13/ km^2^) and the other closely located districts within Maharashtra have also been severely affected that could have promoted higher viral spread, and given the high PRS, they may present higher susceptibility to COVID-19 infection and associated inflammatory responses.

## Discussion

Previous observation of genetic differences in the individuals from different regions of India, and the strong association between genetic variants and COVID-19 illness led us to evaluate the genetics risk for SARS-CoV-2 mediated illness in Indian sub-populations. In this report, we have calculated the PRSs of SNPs in different Indian sub-populations, followed by a regression analysis with COVID-19 related mortality in different districts of India. Our results indicate a significant association between the number of COVID-19-related deaths with the PRS, and thereby can provide support for population specific prioritization in COVID-19 vaccination program.

In a recent GWAS, these SNPs have been robustly associated with critical illness in patients with COVID-19 (Pairo-Castineira et al., 2021). It has also been noted that the individuals belonging to different linguistic lineages in different geographic locations of India exhibit genetic distinctness (Indian Genome Variation Consortium, 2005; Indian Genome Variation Consortium, 2008). Here, our results indicate that these subtle genetic differences can affect their susceptibility to COVID-19 mediated inflammatory organ damage. Our study adds to the existing literature of association between genetics and COVID-19 severity. This vast genetic differentiation among the ethno-linguistic groups suggested that considering the ∼1.4 billion people in India as “Indians”, as one single genetically homogenous group would lead to false positives in association studies and would require taking into account the genetic heterogeneity of the Indians. Given the lack of such studies in Indian populations, our report forms a strong foundation for future studies, and could aid in identifying the “at-risk” populations, in making drug and dosage interventions, and predisposition maps for Indian sub-populations as was aimed by the IGVC (Indian Genome Variation Consortium, 2005; Indian Genome Variation Consortium, 2008). Our results present an indication of individuals in Indian sub-populations that are at a high risk of developing critical illness due to COVID-19. Since here we are using the associations derived from a majority European individuals and a few South Asian, East Asian and African individuals to study the risk in the Indian populations, the effect sizes from the GWAS would be ideal to use if the LD pattern around the SNPs used are conserved between the populations. In line, we found conservation of LD structure across populations, and this observation further strengthens our results. These genetic risk scores can, in turn, be employed as a basis of further management of COVID-19 and in the COVID-19 vaccination disbursal scheme. Also, a similar trend was observed in the number of cases over several months in the populations, suggesting that there could be a genetic basis for this trend (Supplementary Figure S3). In the current study, we have also modelled the effect of potential confounders such as population density, age and gender that could also affect the COVID-19 spread and mortality. However, the confounders had only negligible effects in our GLM, reflecting an association between PRS and mortality.

There are certain limitations of the present study. The GWAS was not directly conducted in the individuals of the Indian sub-populations, and the PRSs were based on effect sizes from different ancestral groups with COVID-19 infection. The mortality may also be affected by comorbidities like diabetes, hypertension, cardiovascular diseases (Guo et al., 2020; Yang et al., 2020; McGurnaghan et al., 2021), environmental risk factors and socio-economic factors like multidimensional poverty index (MPI) that can act as confounders. Availability of such information can allow the study of their effects in such models. Since data for age and sex of the affected patients were not available, we employed census data. However, using patient-specific information to model for confounders would have yielded more accurate results. The prediction accuracy can be improved by using sequencing data and since IGVC is array data, some of the top causal variants were not represented which could possibly affect the PRS predictions. A larger sample size could also provide better accuracy, since IGVC captured only a few individuals of each ancestral group.

## Conclusion

In this study, we provide a methodological framework for predicting Indian sub-populations that could be at a higher risk for developing COVID-19 mediated critical illness but not any clinical evidence. These scores in conjunction with the commonly noted comorbidities could provide a good prediction in ascertaining high COVID-19 risk groups. Such accurate identification of vulnerable populations is crucial for the development of effective prevention and vaccination strategies. Such strategies applied to populations with defined genetic histories such as in the Indian subcontinent can be easily extended to model population level susceptibility to several other important diseases that strain the public health system in India, and provide a necessary use case justifying national scale projects such as GenomeIndia.

## Data Availability

The datasets presented in this study can be found in online repositories. The names of the repository/repositories can be found below: For all our analyses, we used the summary statistics available at https://genomicc.org/data, also provided in the publication of the GWAS used. The COVID-19 data for the Indian population was retrieved from ftp://ftp-trace.ncbi.nih.gov/1000genomes/ftp/release/20130502/, https://www.covid19india.org/, https://covid19.Assam. gov.in/district/, https://coronaclusters.in/telangana/warangal-urban and https://github.com/covid19india/api. The population density, age and sex data for the districts studied were collected from https://censusindia.gov.in. The codes and data of the 100 SNPs used for the study can be accessed at the following GitHub repository: https://github.com/Prakrithi-P/COVID_PRS_IGV. The genotype data from IGV Consortium is an in house dataset and is not publicly available. Requests to access this should be directed to MM: mitali@igib.res.in, mitali@iitj.ac.in or at http://igvdb.res.in/.
