# Supplementary material for: Genetic Risk Prediction of COVID-19 Susceptibility and Severity in the Indian Population
Source: Front Genet. 2021 Oct 11;12:714185. doi: 10.3389/fgene.2021.714185 (PMC8543005; doi:10.3389/fgene.2021.714185)
Supplement: Supplementary file 2 [file DataSheet1.docx]

### **Supplementary Figures**

### **
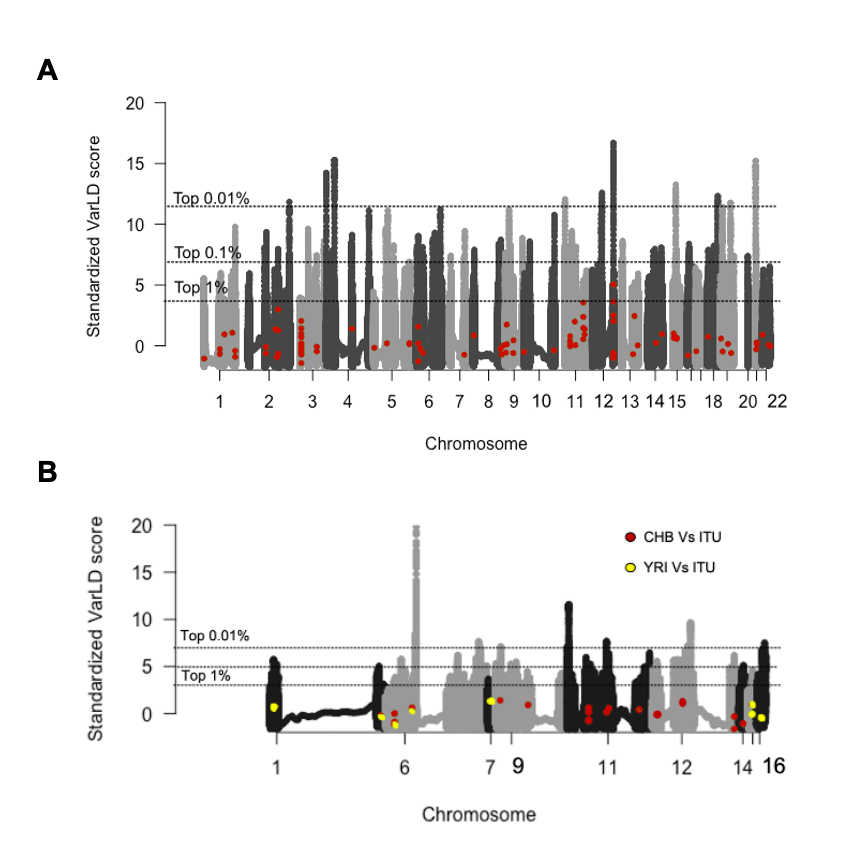
**

**Supplementary Figure S1 (A)** Standardized varLD score across CEU and ITU populations. varLD scores for the SNPs analyzed in this study are marked in red, and majority of these are located in the low varLD regions reflecting low differences in LD with respect to these SNPs in these two populations. The dotted lines indicate the VarLD thresholds. Regions above the thresholds have high LD differentiation between the compared populations. A similar pattern was observed for the few SNPs whose effect sizes were derived from East-Asian and African ancestral populations with (B) CHB vs ITU (red) and YRI vs ITU (yellow) respectively.


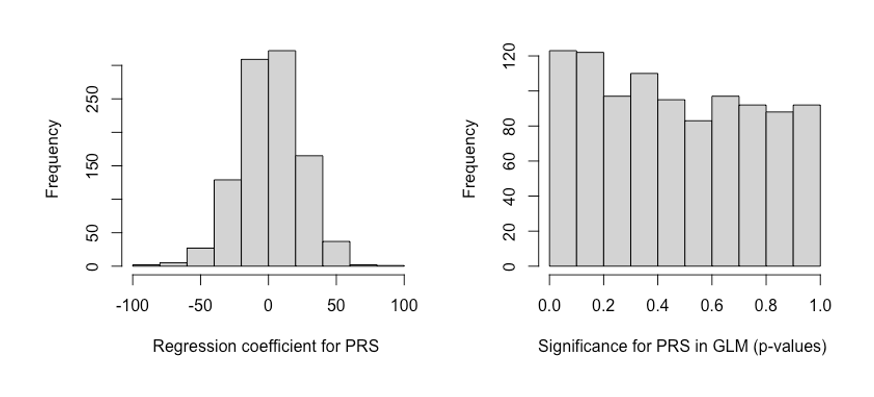


**Supplementary Figure S2** Coefficient and *p*-value estimates in GLMs between COVID-19 mediated deaths and polygenic risk score calculated from 1000 sets of random non-risk SNPs. The *p*-value histogram shows an overall insignificant association.


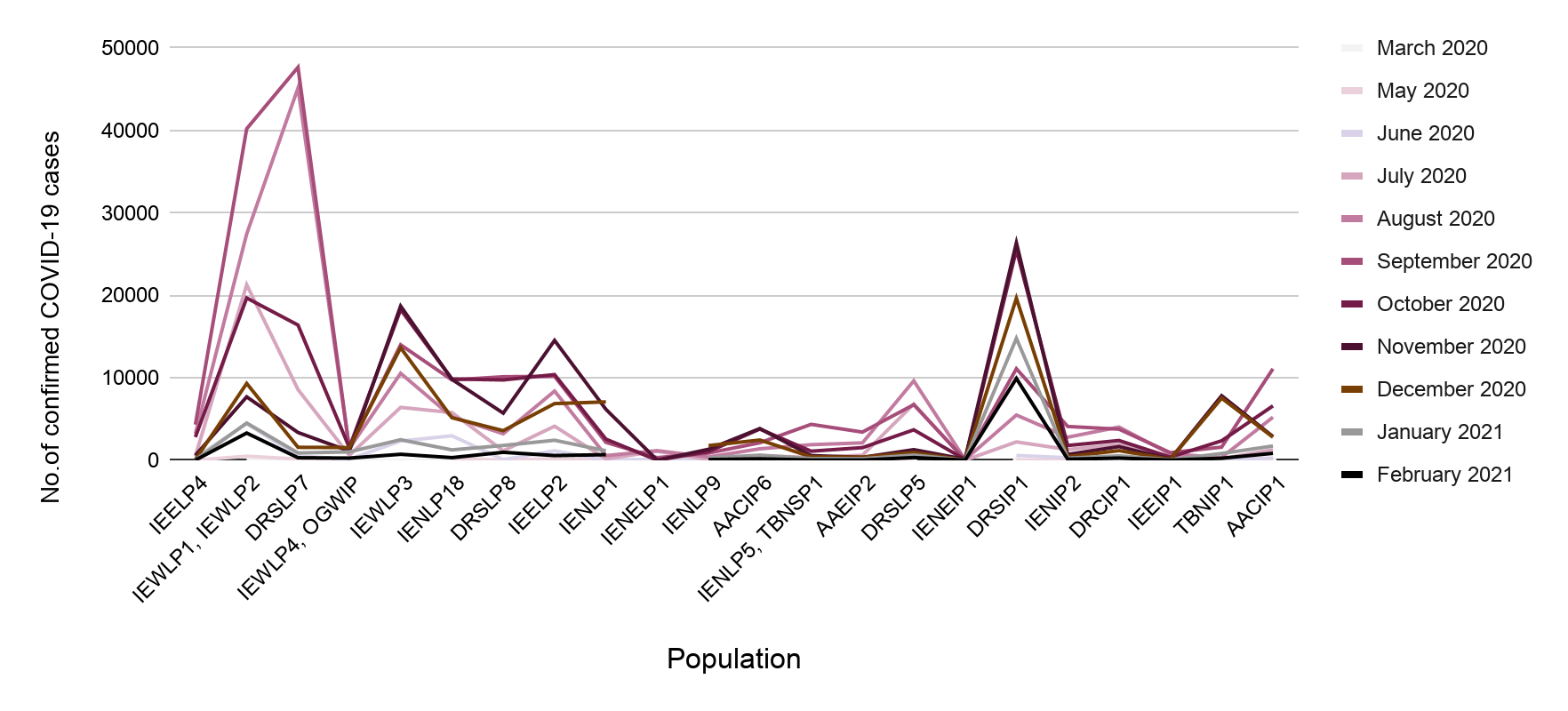


**Supplementary Figure S3** Number of confirmed cases corresponding to different IGVC populations over different months. The populations are ordered by those with highest to lowest PRSs.
